# Ultrafast z-scanning for high-efficiency laser micro-machining

**DOI:** 10.1038/lsa.2017.181

**Published:** 2018-04-20

**Authors:** Ting-Hsuan Chen, Romain Fardel, Craig B Arnold

**Affiliations:** 1 Department of Mechanical and Aerospace Engineering, and Princeton Institute for the Science and Technology of Materials, Princeton University, Princeton, NJ 08544, USA

**Keywords:** high-efficiency micro-machining, laser material processing, TAG lens, ultrafast z-scanning

## Abstract

High-throughput laser micro-machining demands precise control of the laser beam position to achieve optimal efficiency, but existing methods can be both time-consuming and cost-prohibitive. In this paper, we demonstrate a new high-throughput micro-machining technique based on rapidly scanning the laser focal point along the optical axis using an acoustically driven variable focal length lens. Our results show that this scanning method enables higher machining rates over a range of defocus distances and that the effect becomes more significant as the laser energy is increased. In a specific example of silicon, we achieve a nearly threefold increase in the machining rate, while maintaining sharp side walls and a small spot size. This method has great potential for improving the micro-machining efficiency of conventional systems and also opens the door to applying laser machining to workpieces with uneven topography that have been traditionally difficult to process.

## Introduction

The ability to laser machine materials with high resolution and high throughput is critical in advanced manufacturing for a vast array of applications, from photovoltaic cells to bio-compatible micro-components^1, 2, 3, 4, 5, 6, 7, 8, 9, 10, 11, 12, 13, 14, 15^. The precision of these manufacturing techniques relies on focusing a laser beam to a micron-sized spot onto the surface of the workpiece. This tight lateral focusing comes at a price since it narrows the depth of field (DOF) along the axial direction, causing a concomitant drop in machining efficiency outside the reduced range. Therefore, the surface of the workpiece has to be carefully maintained within the focal position of the laser beam to ensure a high efficiency; as material is removed, it is important to continue to adjust the location of the laser focus to follow the change in topography of the workpiece^16^.

One common method used to mitigate a narrow vertical machining range consists of extending the DOF of the system by using, for example, a low focusing power lens or structured light. However, such attempts lead to a significant loss in lateral resolution^17^. Alternatively, the focused beam can be adjusted with respect to the surface location throughout the material removal process, but this requires real-time knowledge of the surface location as well as a fast focusing method. While some studies have attempted to acquire real-time surface location information to increase the machining efficiency, the complexity and inflexibility of the resulting machining system drastically reduces its potential for practical use^18^. In addition to the difficulty of real-time surface monitoring, most controlled focusing methods also suffer from low response rates compared to the repetition rate of the laser^16, 18, 19, 20, 21, 22, 23, 24, 25^.

Here, we demonstrate a new method for increasing the micro-machining efficiency by combining a high repetition rate laser with an ultrafast z-scanner. This creates an axial distribution for the focused beam that allows the pulses to be focused onto different surface locations of the material without real-time monitoring. More importantly, the extended range for focal positions also relaxes the constraints on surface flatness and positioning. The method shown in this paper represents a promising advance in high-efficiency micro-machining and provides great potential for laser machining in a broad range of materials.

## Materials and methods

**Figure 1:**
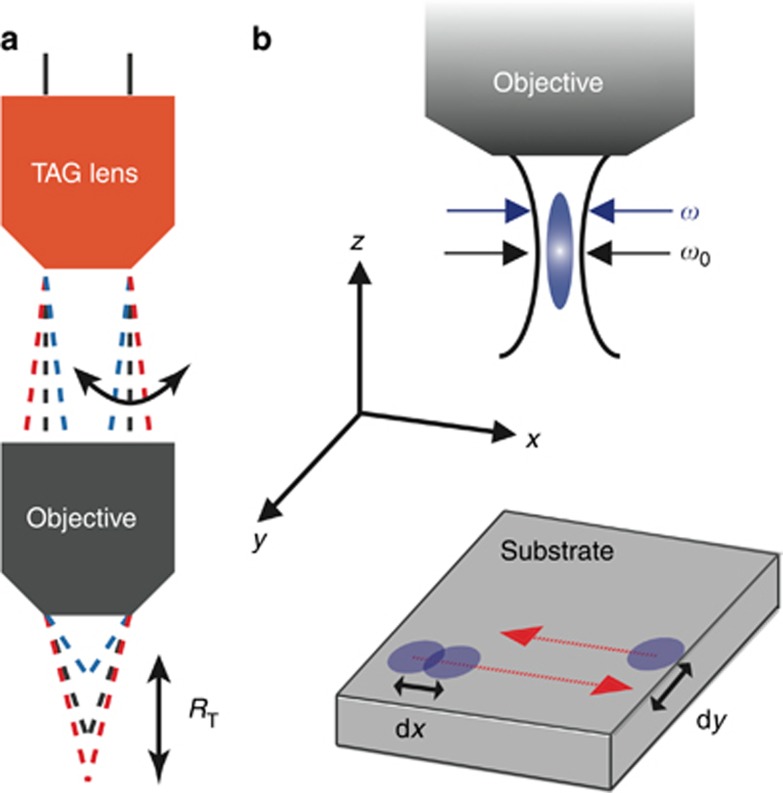
(**a**) Experimental setup of the system. The laser beam is guided through the TAG lens and objective before being focused onto the substrate. The TAG lens is an ultrafast vari-focal device that generates sine wave oscillations in lens power (a quantity that is linearly dependent on the driving voltage). These oscillations result in variations in the focal position below the objective, which we call the laser scanning range, or *R*_T_. (**b**) In this paper, we adopt the convention that the laser beam travels in the negative *z* direction. The beam waist is shown as a curved black line; the position at which the beam waist is narrowest defines the focal point. Ablation occurs when the fluence is larger than the threshold fluence. The blue circle indicates the region in which ablation occurs: the ablation range. The bottom diagram shows the line scan path during the milling experiment, where the blue circles represent the focused beam location and the red lines indicate the beam path along the substrate.

The experimental setup is shown in Figure 1a. A Nd:YVO_4_ laser (Coherent, Inc., Santa Clara, CA, USA, 355 nm, 15 ns pulse duration) is guided through a Tunable Acoustic Gradient-Index (TAG) lens (TAG Optics, Inc.) and focused onto the substrate, which is in our case a 500-μm-thick silicon wafer, using a microscope objective (5 ×, N.A. 0.13). The TAG lens is an ultrafast vari-focal device that has a lens power (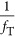, *f*_T_ is the focal length of TAG lens) that varies sinusoidally, with the oscillation frequency set to 140 kHz^26^. This results in an adjustable focus position after the objective, which is indicated as the scanning range, *R*_T_, in Figure 1a. The scanning range is simulated in Zemax (Zemax LLC) using the TAG lens model detailed in Ref. 27. Table 1 shows a few relevant scanning ranges.

For the silicon experiment, we machine a nominal 200 μm by 200 μm square hole with and without z-scanning. The silicon thickness is 500 μm. The spacing between pulses is 1 μm, and the laser repetition rate is 1 kHz. Each pass is accomplished by bi-directional line scanning over the entire square, as in Figure 1b, with 175 to 200 pulses per line, depending on the spot size.

A similar setup is utilized in experiments carried out for Kapton with a thickness of 135 μm. A Nd:YAG laser (EKSPLA, 355 nm, 30 ps) and a 15 × microscope objective lens (N.A. 0.32) is used in this case. The different materials machined under various scanning ranges are examined and characterized under a scanning electron microscope and a laser scanning confocal microscope (Olympus LEXT).

## Results and discussion

### Silicon machining results

**Figure 2:**
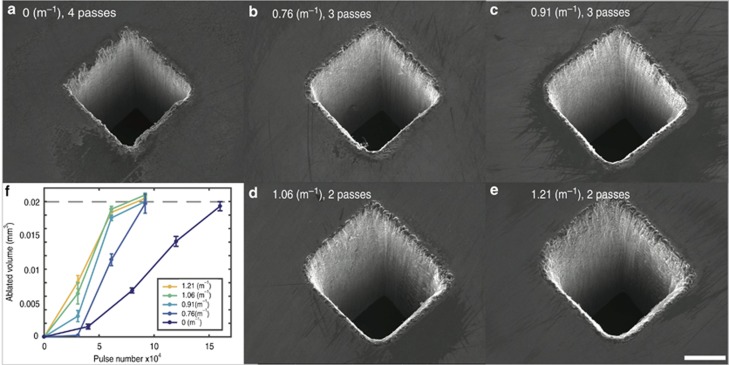
(**a**) SEM images of a square hole ablated without a z-scanner (lens power of 0 m^−1^). The hole is a 200 μm by 200 μm square with a depth of 500 μm. Without z-scanning, we require four passes over the substrate to ablate the desired volume. However, we require only three passes with lens powers of (**b**) 0.76 m^−1^ and (**c**) 0.91 m^−1^. When we further increase the lens power to (**d**) 1.06 m^−1^ and (**e**) 1.21 m^−1^, 90% of the desired ablation is completed in only two passes. The measured ablated volume versus pulse number is shown in (**f**). The scale bar shown corresponds to 100 μm.

We machine a 200 μm by 200 μm square hole with and without z-scanning while maintaining a constant laser peak fluence of 500 J cm^−2^. Without scanning, four passes are required to completely machine the square hole, as shown in Figure 2a. This results in an average machining rate of 9.04 × 10^8^ mm^3^ per pulse. After enabling the z-scanner at a lens power of 0.91 m^−1^, the machining is ~88 % complete after two passes and 100 % complete after three passes, as shown in Figure 2b and 2c. Increasing the lens power to 1.06 m^−1^ or 1.21 m^−1^, we find that 90 % of the machining is complete after two passes and is fully complete after three, as shown in Figure 2d and 2e. The measured ablated volume versus pulse number is shown in Figure 2f. This corresponds to an increased rate of 2.50 × 10^7^ mm^3^ per pulse at a lens power of 1.06 m^−1^; therefore, the machining rate is more than doubled with the assistance of the ultrafast z-scanner.

This increase in machining rate is a highly promising result, but such gains are often accompanied by degradation of the machining quality, i.e. a poor lateral resolution and wall angle. Variations in the lateral resolution are directly related to the smallest spot size that the laser can produce on the substrate. With z-scanning enabled, the spot size is predicted to be larger: in this specific case, three times larger than that achieved without scanning due to the fluence being 500 times larger than the threshold fluence^28^. Such a minor degradation in resolution is acceptable in most of the aforementioned applications.

**Figure 3:**
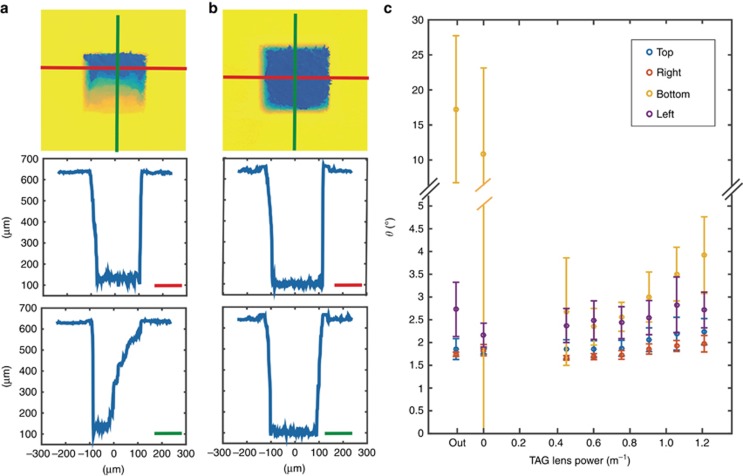
(**a**) To compare machining quality, we ablate a 200 μm by 200 μm square on a 500-μm-thick silicon wafer with three passes at a lens power of (**a**) 0 m^−1^ (no z-scanning) and (**b**) 0.91 m^−1^. The second row of (**a** and **b**) shows the horizontal cross-sectional profiles, as indicated by red lines in the first row. The third row similarly shows the vertical cross-sectional profiles, as indicated by green lines in the first row. In (**c**), we plot the wall angles as a function of the lens power, where the wall angle is a measure of the deviation from the desired right angle. Clearly, for a fixed number of passes, the addition of z-scanning produces a much more uniform wall. In addition, deviations grow slowly with increasing lens power.

To investigate the wall angle produced by our system, we machine a 200 μm by 200 μm square on silicon at different lens powers. Figure 3a and 3b shows the machining results obtained for three passes at lens powers of 0 and 0.91 m^−1^. The horizontal and vertical cross-sectional profiles of the square holes are shown in the second and third rows of Figure 3a and 3b. The difference between the horizontal and vertical cross-sectional profiles of the square hole can be caused by the re-deposition of materials^29, 30, 31^. The wall angles corresponding to each side of the square are measured separately and are shown in Figure 3c as angular deviations from the desired right angle. The data show that this discrepancy increases slightly at higher lens powers; however, the discrepancies are more uniform compared to the case for zero lens power and never exceed five degrees. Therefore, we doubled the machining efficiency of the system with a minimal loss in the quality of the lateral resolution and wall angle.

**Figure 4:**
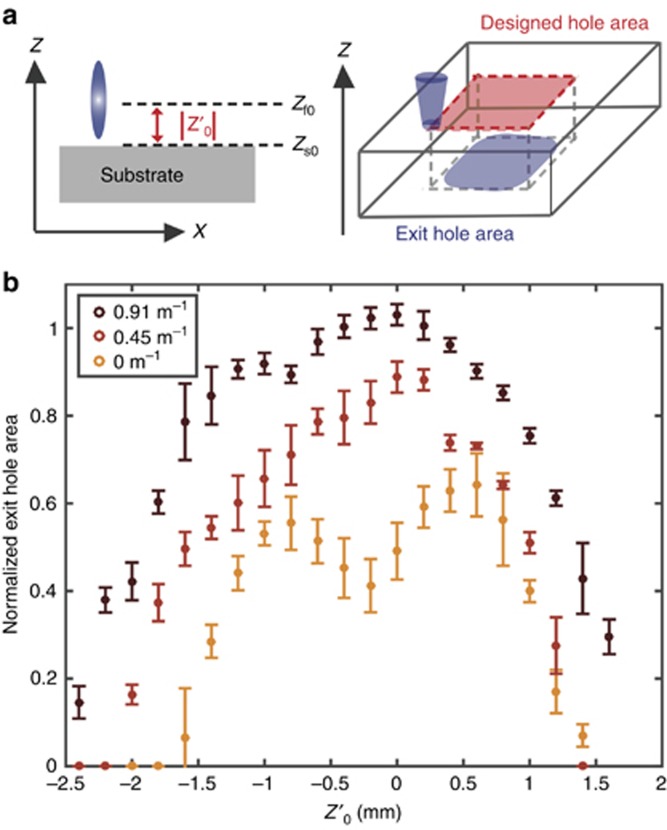
(**a**) The surface of the substrate at *z*_s0_ is placed at a fixed distance from the focal point at *z*_f0_ throughout the machining process. We refer to the difference in these quantities as the defocus distance and measure the exit-hole area at three different lens powers and over a range of 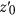 values (three passes were used in all cases). The results shown in (**b**) are normalized by the designed hole area and are shown with error bars determined from ten different experiments.

The benefits of z-scanning extend beyond an increased machining efficiency. As our system scans through different focal positions, the laser pulse reaches different axial positions at the surface, which enables uniform machining over a range of axial substrate positions. This suggests that similar machining efficiencies can be achieved even for a non-flat surface. To test our hypothesis, we place the sample at a fixed defocus distance, 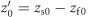, which is the distance between the focal position, *z*_f0_, and the surface, *z*_s0_, without z-scanning, and machine a square, as before, at three different lens powers. The process is illustrated in Figure 4a. We then measure the normalized exit-hole area, noting that a value of one indicates ideal performance. A plot of this area over a range of 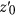 values and at three different lens power levels is shown in Figure 4b. Our hypothesis implies that at a higher lens power, we should obtain similar machining results over a range of 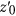 values. This behavior is confirmed in Figure 4b. This means that z-scanning effectively relaxes tight focusing constraints, which is particularly beneficial for samples with rough surfaces, such as those found in most real-world materials.

So far, we have compared the machining results from fast scanning to those from a fixed focus. However, one can hypothesize that a stepwise focal adjustment after each machining pass could further improve the material removal rates. We consider this method in the Supplementary Information (Supplementary Fig. S1) and find that it is not as efficient as the fast scanning method. The reason for this inefficiency is that stepwise machining assumes perfect knowledge of the surface location, which is not necessarily uniform because of material re-deposition and other phenomena. Use of our new method results in significantly increased machining rates at all defocus distances, with a negligible loss of product quality, and further enables efficient machining on non-flat surfaces that would have otherwise required expensive and slow focus control systems.

### Ablation rate analysis

#### *Non-scanning*

To explain how focus scanning improves the machining efficiency and enables an extended machining range in the *z* direction, we calculate the machining efficiency as a function of the defocus position, 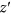. The efficiency of a micro-machining system is directly related to the amount of material removed by a single laser pulse, which we call the single-shot ablated volume, or *V*_ss_. This value can be expressed as the integral of the ablation depth, *D*, over the ablated area, *A*:


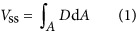


The ablation depth can be derived based on knowledge of specific material and laser properties. In our case, we assume that the Beer–Lambert model holds, so


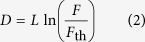


where *F* is the laser fluence, *L* is the effective laser penetration depth, and *F*_th_ is the threshold fluence of the material. Although *L* and *F*_th_ are measured experimentally, as detailed in the Supplementary Information, we must still determine an expression for *F*. The laser is a Gaussian beam, which for a fixed focal position, *z*_f_, causes the laser fluence to take on the following functional form:


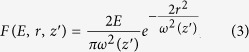


where 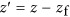 is the defocus distance measured from the focal position, 
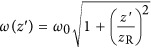
 is the beam diameter at a particular *z* position, 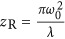 is the Rayleigh length, *E* is the pulse energy, and *r* is the radial coordinate in the *x*–*y* plane. The beam waist, *ω*_0_, is measured by experiments detailed in the Supplemental Information^32^. By substituting Equation (3) into Equation (2), we find


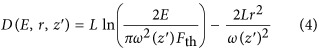


The ablation depth is a function of *r*^2^, indicating that the ablation hole will form a paraboloid. As the volume of a paraboloid has a simple analytic expression, we can rewrite Equation (1) as


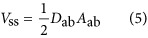


where *D*_ab_ is the depth of the hole and *A*_ab_ is the base area. We find *D*_ab_ by setting *r*=0, in Equation (4),


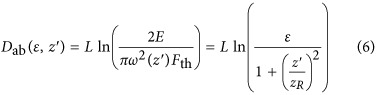


where we have introduced the non-dimensionalized pulse energy 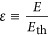, where 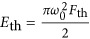 is the threshold energy required for ablation at the focal point. Turning to *A*_ab_ in Equation (5), we have


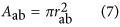


where the radius *r*_ab_ can be found by solving 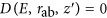. The latter gives


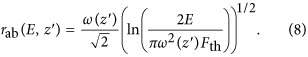


Thus, the base area is


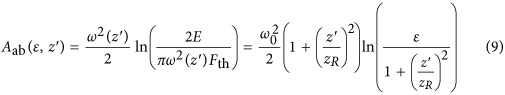


Finally, combining Equations (6) and (9) in Equation (5) we find,


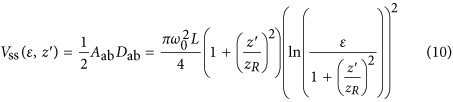


**Figure 5:**
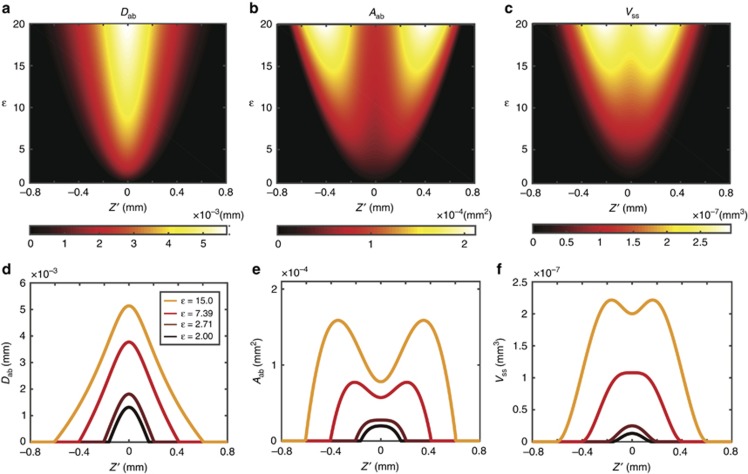
(**a**) The central depth of the single-shot ablated hole is plotted as a function of the energy level, *ε*, and defocus distance, 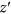. Similarly, the base area of the ablated hole, *A*_ab_, is plotted in (**b**). The volume of the ablated hole, *V*_ss_, can then be calculated with knowledge of the central depth and the base area of the ablated hole, as shown in (**c**). To illustrate the ablated volume profile change, we select four *ε* to represent different cases and plot the corresponding *D*_ab_, *A*_ab_ and *V*_ss_ in (**d**–**f**), respectively.

In Figure 5a–5c, we plot, from left to right *D*_ab_, *A*_ab_, and *V*_ss_ as functions of 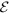 and 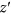. We can see from Figure 5c that the maximum *V*_ss_ is located at the origin of 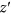 for smaller values of *ε*, while it splits at higher energy levels. This implies that the maximum ablation rate can be achieved by defocusing at some higher energy level. We find the defocus distance at which the ablated volume *V*_ss_ has a maximum by taking the derivative with respect to the defocus distance 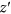. We provide detailed calculation in the Supplementary Information. The maximum *V*_ss_, *V*_m_, occurs at 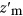, such that


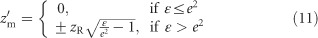


leading to the expressions,


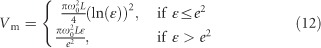


We pick four different energy levels for *D*_ab_, *A*_ab_ and *V*_ss_ and plot them as a function of 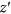 in Figure 5d–5f, respectively. We can see that *D*_ab_ monotonically increases as the energy level increases, while the profile of *A*_ab_ changes from a unimodal profile to a bimodal profile at energy level *ε*=*e*, as discussed in our previous work^28^. Interestingly, when these curves are combined to form V_ss_, we find that the transition point shifts to *ε*=*e*^2^. Above this value, the maximum material removal rate is not located at 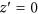, implying again that a greater ablated volume can be achieved by purposefully defocusing the laser.

#### *Scanning*

So far, we have discussed the case for a fixed focus system (*z*_f_=*z*_f0_). We now incorporate into our analysis of the effect of the variable focal position introduced by the TAG lens. The focal position varies sinusoidally, as described in Materials and Methods:


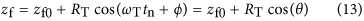


*t*_n_ is the reciprocal of the laser repetition rate, which is constant during the experiment. *ω*_T_ is the driving frequency and varies by ~1% throughout the experiment, and *Φ* is the phase, which takes a new value for each new line. The equation is simplified by grouping terms into *θ*=*ω*_T_*t*_n_+*Φ*, which accounts for the variations in focal positions.

Since the ablation rate is a function of 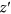, i.e. the distance between the focal position and surface, as discussed in Equation (10), the ablation rate can be calculated by considering the probability distribution (PDF) of the focal position, *p*(*z*_f_), by assuming a fixed surface position. For a given set of parameters, *R*_T_ and *z*_f0_, the PDF of the focal position can be determined by knowing the probability distribution of *θ*, *p*(*θ*), and basic formulae for the probability transformations^33^. This gives


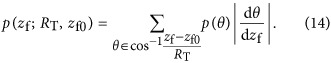


In the case of a statistically representative number of pulses, such as in our experiments, *θ* will assume all possible values between 0 to 2*π* with equal probability. Thus, we express the probability of *θ* as


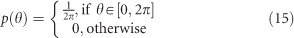


In the range of [−*R*_T_, *R*_T_], there are two solutions to the equation *z*_f_=*z*_f0_+*R*_T_cos(*θ*) for *θ*∈[0, *π*]: one in [0, *π*] and the other in [*π*, 2*π*]. Thus


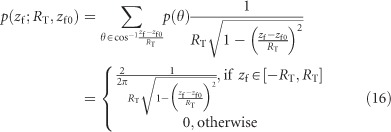


**Figure 6:**
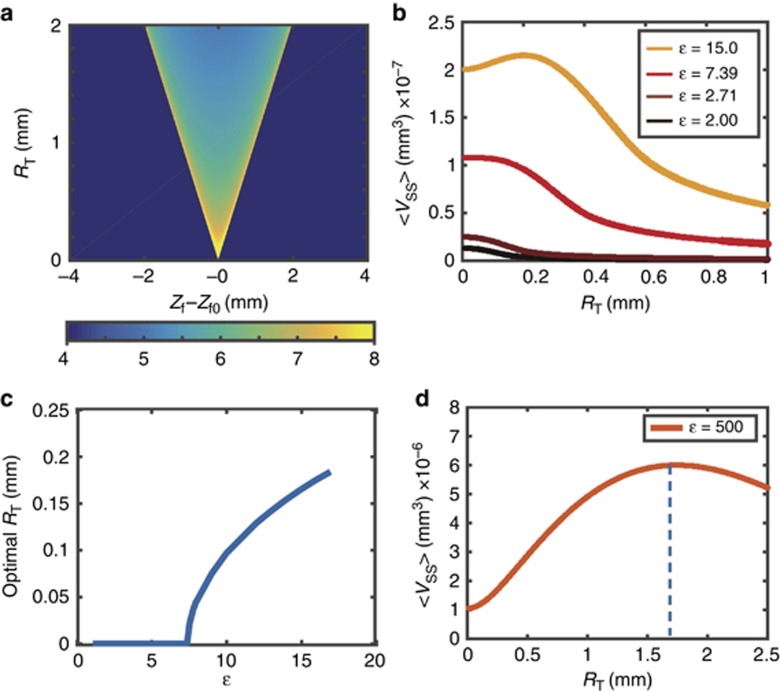
The PDF of the pulse positions is normalized for each *R*_T_ and plotted in (**a**). We then calculate the expected ablated volume, 〈*V*_ss_〉, by using *V*_ss_ and the PDF of the focal position for each scanning range, as plotted in (**b**). The scanning range that maximizes the ablated volume, optimal *R*_T_, is then calculated and plotted in (**c**). To compare with our silicon experiment, we plot the expected ablated volume as a function of scanning range, which shows the optimal scanning range is at 1.74 mm, as marked by the blue dashed line. This result is close to that used in our experimental conditions.

The PDF of the focal position is plotted in Figure 6a. From Equation (10), we see that the material removal rate is dependent on the defocus distance, 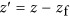, which is the distance between the focal position and surface. In our model, the focal position is a probability function of the scanning range, as described in Equation (16). Thus, we can calculate the expected material removal rate for a given scanning range through


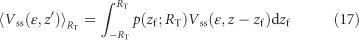


If we assume that the sample is placed at the focal point of the fixed focus system (*z*=*z*_f0_, and, therefore *z*−*z*_f_=−*R*_T_cos(*θ*)), the resulting plot of 〈*V*_ss_〉 at different scanning ranges is given in Figure 6b. The maximum 〈*V*_ss_〉 is located at *R*_T_=0 in the small energy regime. However, the maximum 〈*V*_ss_〉 is shifted to higher scanning range in the large energy regime. This implies that in the small energy regime, z-scanning is unfavorable; however, in the large energy regime, z-scanning should serve to increase the maximum material removal rate.

We then calculate the scanning range that maximizes the ablated volume at each energy level and plot the results in Figure 6c. Here, we can clearly see that a scanning range of zero is favored when *ε*≤*e*^2^, where *V*_ss_ has a unimodal profile. When *ε*>*e*^2^, *V*_ss_ takes on a bimodal profile, and one can improve the material removal rate through the addition of z-scanning at a well-defined scanning range, as shown in Figure 6c. Interestingly, the optimal scanning range takes the same value as the defocus distance that maximizes the ablated volume, 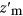, as described in Equation (11). This finding explains why extending the z-scanning range to 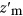 enables one to achieve an optimal ablation rate at the surface. Since the maximum ablation rate increases linearly with laser energy, the enhancement in the machining rate provided by z-scanning is expected to increase with increasing pulse energy, as described in Equation (12). However, this gain in the machining rate would be unavoidably accompanied by an increase in spot size. Duocastella *et al.* has reported a loss in lateral resolution when the energy level is larger than *e*, which is always the case for this method, with this loss increasing with the energy level^28^. Therefore, one should carefully balance these factors according to the demands of each particular application.

We now compare the predicted optimal *R*_T_ with the silicon machining experimental conditions provided in Table 1. The expected ablated volume at different scanning ranges is plotted in Figure 6d, where we can see that the maximum removal rate is achieved at a scanning range of 1.74 mm, as marked by a dashed line in the figure. This is close to the conditions used for which we observed the best experimental results, which shows that our model provides a useful framework for understanding the general concepts at play.

### Quantitative comparison

So far, we have qualitatively shown that scanning provides a benefit for improving the machining rate. However, quantitative prediction of the ablated volume in silicon is difficult due to the complex ablation mechanism involved^29, 30, 31^. To make a quantitative comparison between theory and experiments, we focus on a model system, Kapton, whose ablation mechanism follows a simple photochemical desorption model^34, 35^.

**Figure 7:**
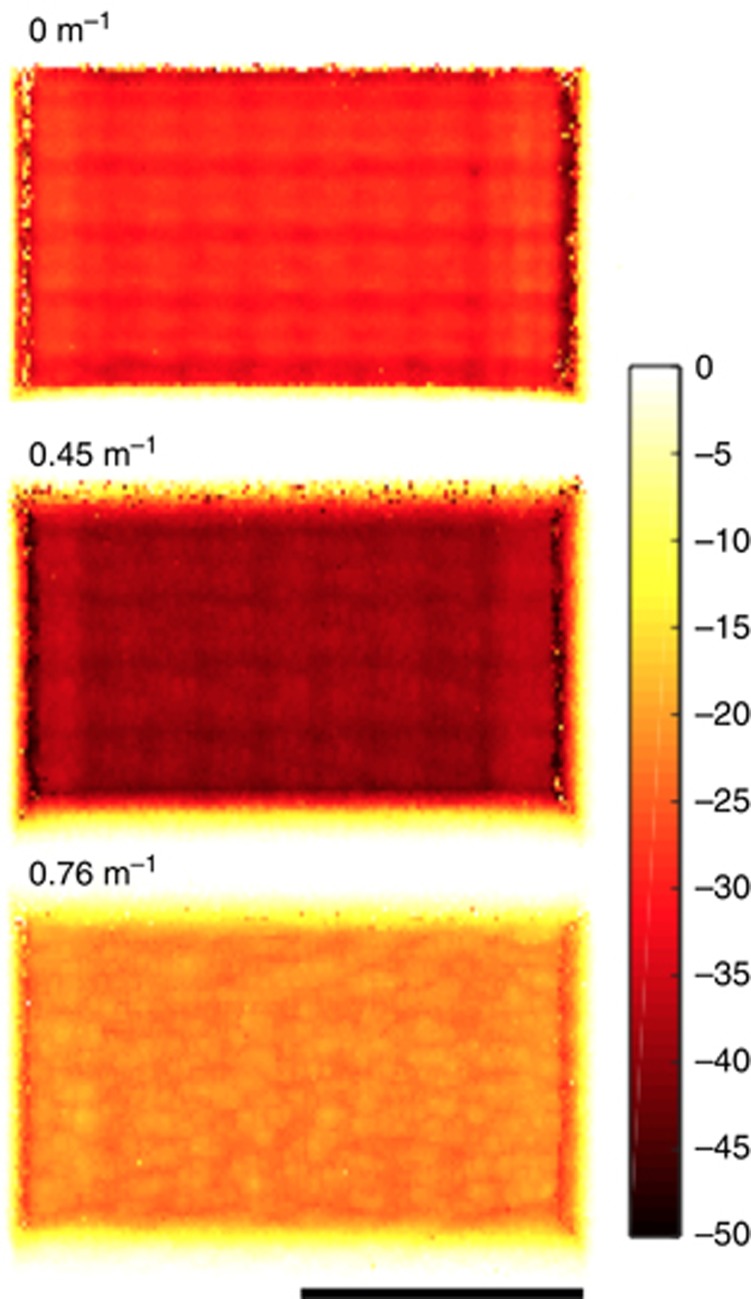
The depth profile of the pocket machined in Kapton using different lens powers. All of these profiles are machined at a defocus distance 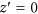. The unit for the color bar is μm and for the scale bar is 100 μm.

**Figure 8:**
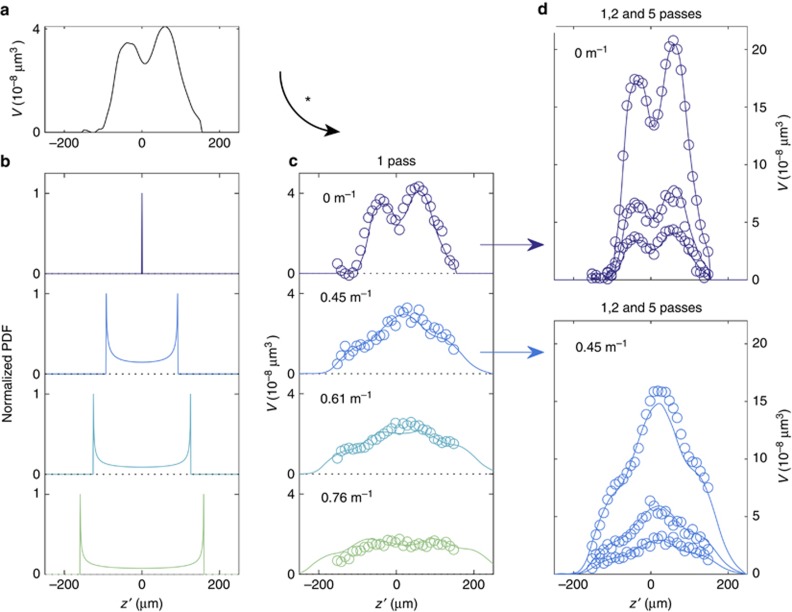
We machine a pocket into Kapton at different defocus positions. The ablated volume as a function of 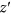 is shown in (**a**). The PDF of the focal positions with lens powers of 0 (m^−1^), 0.45 (m^−1^), 0.61 (m^−1^) and 0.76 (m^−1^) is normalized and shown in the same order from top to bottom in (**b**). The calculated and experimental results for the ablated volume function with different lens powers are shown in (**c**). The calculated data (smooth curve) show good agreement with experiment (scattered points). We further apply linear extrapolation for multiple passes, and find the calculation still shows good agreement with the experimental data, as shown in (**d**).

We machine a pocket into Kapton at different defocus positions and scanning ranges, as explained in Materials and methods section and Figure 4a. The profile of the machined pockets at 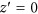 is shown in Figure 7. The laser energy level used is *ε*=*e*^2^. We measure the ablated volume over a 100 μm by 100 μm area for each defocus setting. The fixed focus ablated volume as a function of 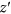, 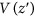 is obtained by interpolating experimental data and is shown in Figure 8a. The asymmetric shape of the ablation depth over the defocus distance could be caused by the intersecting beam divergence, as discussed in the previous literature^36^. The PDF of different scanning ranges is calculated and shown in Figure 8b. Similarly, with knowledge of the ablated volume function *V* and PDF of the focal positions, we can derive the expected ablated volume value, as described in Equation (17). In each subfigure of Figure 8c, we fix the scanning range and vary the defocus distance, calculating the expected ablated volume for each new experiment (scattered points). We compare this to theoretical predictions derived from Equations (16) and (17) (smooth curve) and find excellent quantitative agreement. Figure 8c also confirms our hypothesis from Silicon machining results section that similar machining efficiencies can be attained over a wide range of 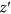 values, especially at a higher lens power. This highlights the other significant benefit of this system in that using a z-scanner not only increases the machining efficiency but also enables an extended machining range in the *z* direction. We achieve a uniform machining rate over a range of 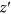 values by using ultrafast z-scanning and therefore eliminate the need for real-time focus control. Figure 8d compares experimental results with theoretical predictions for multiple passes, which again shows strong quantitative agreement. Here, our predicted curves for multiple passes are found simply by scaling the single pass curve by the number of passes. Since the incremental increase in ablation depth is nearly linear for small numbers of pulses, we expect a simple scaling approximation to be valid for our settings.

## Conclusion

To meet the growing demand for micro-machined products, such as photovoltaic cells, electronic devices, and medical micro-elements, we demonstrate a new high-efficiency laser machining method enabled by an ultrafast z-scanner. Machining efficiency can be derived as the material removal rate function, which is heavily dependent on the relative positions of the focal point and substrate surface. To date, efficiency gains have been limited by the difficulty of pairing real-time surface monitoring with requisite tight focus control. Here, we eliminate the need for this type of control by combining rapid focal position scanning with rapid laser firing. This creates a system in which the focal point is no longer at a fixed position, but is rather described by a probability distribution whose values are dependent on the scanning range. Our model predicts that focus scanning will result in an increased machining efficiency when the laser energy is higher than *e*^2^ times the threshold energy and that such improvements will grow as the laser energy further increases. We verify this using the model substrate of Kapton. However, increasing the laser energy for a Gaussian beam can also lead to an increase in the minimum resolution; therefore, one should carefully consider such a trade-off in light of the desired product features.

We further demonstrate our method using the technologically important substrate of silicon. Based on our experimental conditions, our model predicts an optimal scanning range of 1.74 mm, which is close to the optimal experimental conditions. At these settings, we find a nearly threefold increase in machining efficiency compared to a system without z-scanning. The extended range of focal positions not only increases machining efficiency but also relaxes the requirement of an accurate focus control. Thus, the method presented in this paper significantly enhances the efficiency of existing systems, while opening the door to the machining of non-flat materials found in real-world applications.

## Figures and Tables

**Table 1:** The scanning range corresponding to the TAG lens power is simulated in Zemax with 5 × and 15 × objectives

**Lens power (m**^**−1**^)	***R***_**T**_ (**5 ×) (mm)**	***R***_**T**_(**15 ×) (mm)**
0.45	0.73	0.09
0.61	0.99	0.14
0.76	1.24	0.16
0.91	1.51	0.19
1.06	1.80	0.23
1.21	2.09	0.27
