# Supplementary material for: Ultrafast z-scanning for high-efficiency laser micro-machining
Source: Light Sci Appl. 2018 Apr 20;7:17181–. doi: 10.1038/lsa.2017.181 (PMC6060055; doi:10.1038/lsa.2017.181)
Supplement: Supplementary Information [file lsa2017181x1.pdf]

Ultrafast z-scanning for high-efficiency laser micro-machining

Ting-Hsuan Chen, Romain Fardel, and Craig B. Arnold*

Department of Mechanical and Aerospace Engineering, and the Princeton Institute for Science and Technology of Materials, Princeton, New Jersey 08544, USA

E-mail: cbarnold@princeton.edu

Machining experiments on silicon with a fixed focal point and a stepwise moving focal point

To achieve high-efficiency micro-machining, one can place the surface of target material at the optimal machining position with respect to the laser focus. As the material is removed during the machining process, the surface will unavoidably fall out of the optimal machining position. A common method to mitigate this problem is to move the target material or focal position stepwise to maintain the optimal machining position. The process is sketched in figure S1(a). Here we perform an experiment to compare this stepwise machining (SW) strategy with the fixed focus (FF) strategy and the TAG lens (TL) strategy. The machining results by (FF) strategy are shown in (b), (c), (d), and (f). The machining results by the (SW) strategy are shown in (e) and (g) with the (TL) results included in (g). As we hypothesize, the (SW) machining rate is improved relative to (FF). However, we can see that the redeposition of the materials causes an uneven surface depth after machining. This makes it difficult to optimize the (SW) strategy for a given system. In the main text of this

paper, we demonstrated the (TL) strategy, which improves the machining rate by scanning the focal position rapidly. This method not only improves the machining rate but also relaxes the constraints on surface flatness and positioning. As shown in figure S1(g), the ablated volume is maximal for the (TL) machining at all defocus distances compared to different (SW) and (FF) processes.

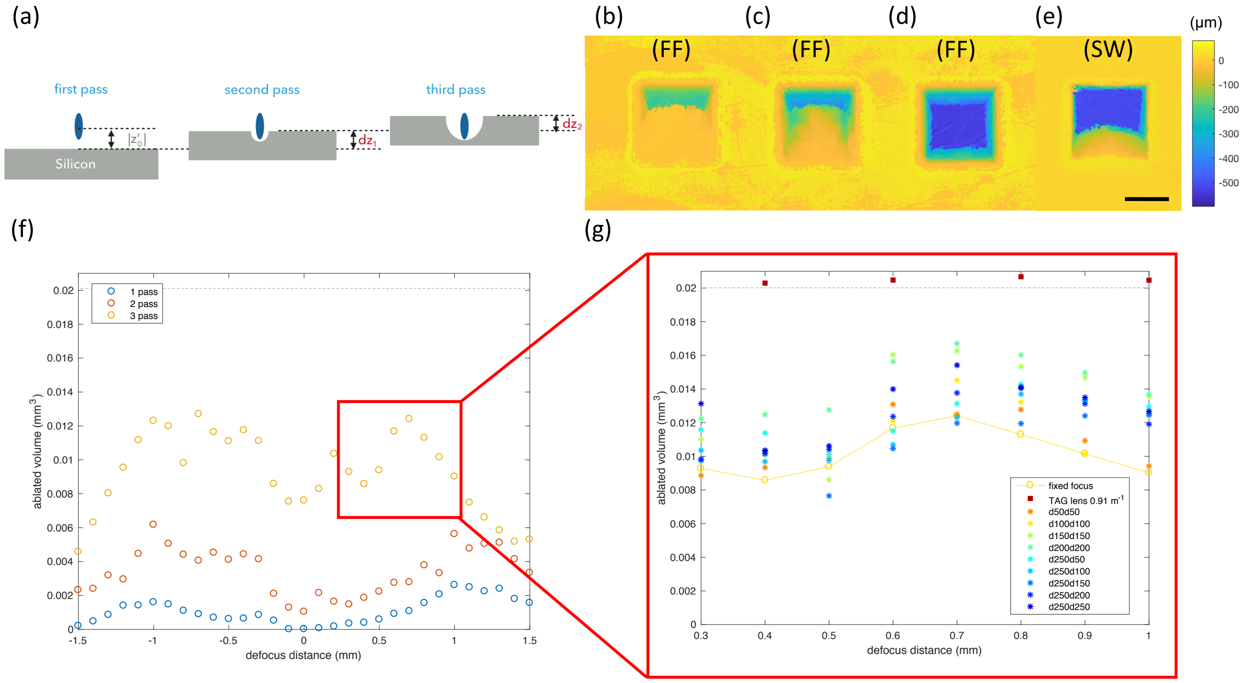

Figure S1: The schematic for stepwise (SW) machining process is shown in (a). The surface of the material is placed at a defocus distance, z_0', away from the focal point in the first pass. The target material is then raised by dz_1 in the second pass and raised again by dz_2 in the third pass. The (SW) strategy is compared with fixed focus (FF) machining. The square machined at a fixed focus $z_0' = 0.7$ mm by one pass, two passes and three passes are shown in (b), (c) and (d). (e) The square machined at $z_0' = 0.7$ mm for the first pass and the target materials is moved up $dz_1 = 200$ μm for the second pass and moved up again $dz_2 = 200$ μm for the third pass. The scale bar shown is 100 μm. We show the ablated volume by (FF) in (f) and the ablated volume by (SW) and (TL) in (g). In the figure (g), the (SW) curves are labeled "*dxxxdyyy*", where *xxx* corresponds to dz_1, in figure S1(a) and *yyy* corresponds to dz_2 in figure S1(a).

Experiments for measuring laser and material parameters

In order to know the beam waist of our Gaussian beam, we perform single shot ablation experiment on silicon at different laser fluences.[1] The silicon is placed at the focal position of the system and ablated by a single laser pulse. The beam waist of the laser beam can be determined by fitting the curve of radius versus the laser energy, shown as figure S2. The beam waist of our system is determined to be 4.28 µm.

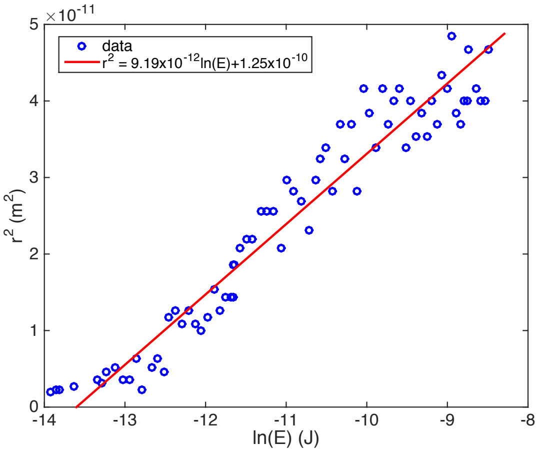

Figure S2: The radius of single shot ablated hole on silicon for different pulse energy are measured and plotted.

Similarly, we perform single shot ablation experiment on silicon at different laser fluences to determine the material related parameters in ablation depth. The central depth of the ablation hole is plotted versus the laser fluence in figure S3. The effective penetration depth of the laser energy $\mathcal{L}$ and the threshold fluence F_{th} of our system is 1.84 µm and 1.0 J cm^{-2}.

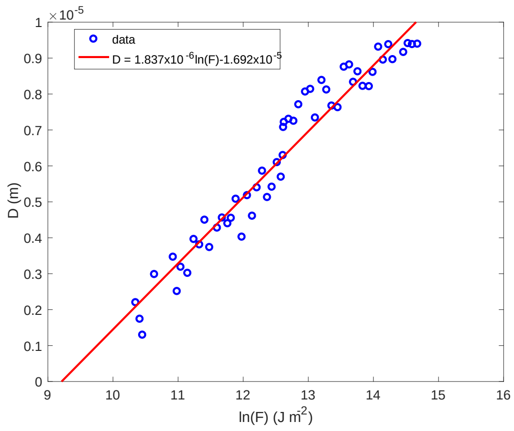

Figure S3: The depth of single shot ablated hole on silicon for different fluence are measured and plotted.

Detailed derivation for transition point of ablation rate profile

In order to find the energy level at which V_{ss} transitions from a unimodal profile to a bimodal profile, we take the first derivative of V_{ss} with respect to z'. The first derivative of V_{ss} with

respect to z' is

$$\begin{aligned}
\frac{dV_{ss}}{dz'} &= \frac{1}{2}(\frac{dA_{ab}}{dz'}D_{ab} + A\frac{dD_{ab}}{dz'}) \\
&= \frac{1}{2}\{[\frac{-\pi\omega_0^2 z'}{z_R^2} - \frac{\pi\omega_0^2 z'}{z_R^2}\ln(\frac{2E}{\pi\omega^2(z')F_{th}})]\mathcal{L}\ln(\frac{2E}{\pi\omega^2(z')F_{th}}) \\
&+ \frac{\pi\omega^2(z')}{2}\ln(\frac{2E}{\pi\omega^2(z')F_{th}})(-\frac{2\mathcal{L}z'}{(1+\frac{z'^2}{z_R^2})z_R^2})\} \\
&= -\frac{\pi\omega_0^2\mathcal{L}z'}{z_R^2}\ln(\frac{2E}{\pi\omega^2(z')F_{th}}) + \frac{\pi\omega_0^2\mathcal{L}z'}{2z_R^2}(\ln(\frac{2E}{\pi\omega^2(z')F_{th}}))^2 \\
&= \frac{\pi\omega_0^2\mathcal{L}}{2z_R^2}z'\ln(\frac{2E}{\pi\omega^2(z')F_{th}})(\ln(\frac{2E}{\pi\omega^2(z')F_{th}}) - 2) \\
&= \frac{\pi\omega_0^2\mathcal{L}}{2z_R^2}z'\ln(\frac{\mathcal{E}}{1+(\frac{z'}{z_R})^2})(\ln(\frac{\mathcal{E}}{1+(\frac{z'}{z_R})^2}) - 2)
\end{aligned} \tag{1}$$

and is equal to zero when $z' = 0$ or $\ln(\frac{\mathcal{E}}{1+(\frac{z'}{z_R})^2}) = 0$ or $\ln(\frac{\mathcal{E}}{1+(\frac{z'}{z_R})^2}) = 2$. The incident fluence must be larger than one for ablation to occur, where $\ln(\frac{\mathcal{E}}{1+(\frac{z'}{z_R})^2}) > 0$. Therefore, The maximum V_{ss} can be found at z'_m that

$$z'_m = \begin{cases} 0, & \text{if } \mathcal{E} \leq e^2 \\ \pm z_R\sqrt{\frac{\mathcal{E}}{e^2} - 1}, & \text{if } \mathcal{E} > e^2 \end{cases} \tag{2}$$

References

(1) Liu, M. J. *Opt. Lett.* **1982**, *7*, 196–198.
